# Effects of Aerobic Exercise, Cognitive and Combined Training on Cognition in Physically Inactive Healthy Late-Middle-Aged Adults: The Projecte Moviment Randomized Controlled Trial

**DOI:** 10.3389/fnagi.2020.590168

**Published:** 2020-10-29

**Authors:** Francesca Roig-Coll, Alba Castells-Sánchez, Noemí Lamonja-Vicente, Pere Torán-Monserrat, Guillem Pera, Alberto García-Molina, José Maria Tormos, Pilar Montero-Alía, Maria Teresa Alzamora, Rosalía Dacosta-Aguayo, Juan José Soriano-Raya, Cynthia Cáceres, Kirk I. Erickson, Maria Mataró

**Affiliations:** ^1^Department of Clinical Psychology and Psychobiology, University of Barcelona, Barcelona, Spain; ^2^Institut de Neurociències, University of Barcelona, Barcelona, Spain; ^3^Institut de Recerca Pediàtrica Hospital Sant Joan de Déu, Esplugues de Llobregat, Spain; ^4^Unitat de Suport a la Recerca Metropolitana Nord, Fundació Institut Universitari per a la recerca a l’Atenció Primària de Salut Jordi Gol i Gurina, Mataró, Spain; ^5^Institut Guttmann, Institut Universitari de Neurorehabilitació, Universitat Autònoma de Barcelona, Barcelona, Spain; ^6^Department of Neurosciences, Hospital Universitari Germans Trias i Pujol, Barcelona, Spain; ^7^Department of Psychology, University of Pittsburgh, Pittsburgh, PA, United States; ^8^Discipline of Exercise Science, College of Science, Health, Engineering and Education, Murdoch University, Perth, WA, Australia

**Keywords:** neuropsychology, lifestyle interventions, computerized cognitive training, physical activity (exercise), aging

## Abstract

**Background:**

Lifestyle interventions are promising strategies to promote cognitive health in aging. Projecte Moviment examines if aerobic exercise (AE), computerized cognitive training (CCT), and their combination (COMB) improves cognition, psychological health, and physical status compared to a control group. We assessed the moderating role of age and sex and the mediating effects of cardiorespiratory fitness (CRF), physical activity (PA), and psychological health on intervention-related cognitive benefits.

**Methods:**

This was a 12-week multi-domain, single-blind, proof-of-concept randomized controlled trial (RCT). 96 healthy adults aged 50–70 years were assigned to AE, CCT, COMB, and a wait-list control group. The per protocol sample, which completed the intervention with a level of adherence > 80%, consisted of 82 participants (62% female; age = 58.38 ± 5.47). We assessed cognition, psychological health, CRF, and energy expenditure in PA at baseline and after the intervention. We regressed change in each outcome on the treatment variables, baseline score, sex, age, and education. We used PROCESS Macro to perform the mediation and moderation analyses.

**Results:**

AE benefited Working Memory (SMD = 0.29, *p* = 0.037) and Attention (SMD = 0.33, *p* = 0.028) including the Attention-Speed (SMD = 0.31, *p* = 0.042) domain, compared to Control. COMB improved Attention (SMD = 0.30, *p* = 0.043), Speed (SMD = 0.30, *p* = 0.044), and the Attention-Speed (SMD = 0.30, *p* = 0.041) domain. CTT group did not show any cognitive change compared to Control. Sportive PA (S-PA) and CRF increased in AE and COMB. Age and sex did not moderate intervention-related cognitive benefits. Change in S-PA, but not in CRF, significantly mediated improvements on Attention-Speed in AE.

**Conclusion:**

A 12-week AE program improved Executive Function and Attention-Speed in healthy late-middle-aged adults. Combining it with CCT did not provide further benefits. Our results add support to the clinical relevance of even short-term AE as an intervention to enhance cognition and highlight the mediating role of change in S-PA in these benefits.

**Clinical Trial Registration:**

www.ClinicalTrials.gov, identifier NCT03123900.

## Introduction

Luckily, most of us are going to age.

Dementia and late-life normal cognitive impairment is related to loss of function and quality of life (Murman, 2015; Christiansen et al., 2019). Therefore, it is a global responsibility to promote healthy aging. *Healthy aging*, conceptualized as “the process of developing and maintaining the functional ability that enables well-being in older age” (World Health Organization [WHO], 2015), has become a social, economic, and scientific challenge. Specific lifestyle behaviors have been associated with better general health (Peel et al., 2005), cognition (Klimova et al., 2017), and well-being (Prendergast et al., 2016) not only in Alzheimer’s disease patients but also in healthy older adults. Evidence shows that exercise and cognitive training may maintain or improve cognition (Kane et al., 2017).

In a meta-analysis in 2003, Colcombe and Kramer reported that aerobic exercise (AE) interventions improved cognitive performance especially for executive function in healthy older adults. Current systematic reviews have replicated those results reporting modest effect sizes of AE interventions on executive function, attention, processing speed, and memory (Smith et al., 2010; Barha et al., 2017; Northey et al., 2018). However, other reviews have questioned the literature and findings of AE interventions on cognition (Young et al., 2015) and refer to significant variation in intervention parameters such as frequency, intensity, time, and type (FITT) to explain heterogeneity across the field. Gomes-Osman et al. (2018) reviewed the relationship between these parameters and positive changes in cognition and concluded that at least 52 h of exercise is required to observe beneficial effects. It is also known that the cognitive effects of AE may be moderated by individual factors such as age and sex (Colcombe and Kramer, 2003; Barha et al., 2017) that are sometimes understudied in randomized controlled trials (RCTs). Other studies have focused on the effects of AE interventions on psychological health and daily activities. Stillman et al. (2016) concluded that increased physical activity (PA) is associated with improved sleep quality and mood. Moreover, AE promotes cardiovascular health by increasing cardiorespiratory fitness (CRF) (Schroeder et al., 2019), which has been linked to brain and cognitive health. However, one remaining question is the mediating role of psychological health and CRF in the cognitive benefits related to AE interventions (Etnier et al., 2006; Young et al., 2015).

Cognitive training has also been associated with cognitive gains in healthy older adults (Ball et al., 2002). Systematic reviews and meta-analysis reported that computerized cognitive training (CCT) interventions are an effective tool to improve memory, processing speed, and visual spatial ability (Lampit et al., 2014; Shao et al., 2015). CCT is less efficacious for attention and executive function (Lampit et al., 2014; Shao et al., 2015). However, one of the main challenges is to assess the degree of transfer between the trained task and untrained tasks (Nguyen et al., 2019). FITT parameters of CCT might be another issue related to inconsistencies in the field. For example, Lampit et al. (2014) found no significant effects when sessions lasted less than 30 min and frequency was more than three sessions of training per week, whereas Chiu et al. (2017) showed greater effectiveness with ≧3 sessions per week and ≧24 total training sessions. As suggested in the literature (Nguyen et al., 2019), individual difference variables such as age could also moderate CCT-related cognitive changes. Other studies have also studied the effect of CCT to improve psychological and self-perceived performance in daily activities. For example, the ACTIVE study found less decline in quality of life for the processing-speed training group. However, memory or reasoning CCT groups did not show any significant change (Wolinsky et al., 2006). Therefore, more research is warranted to better understand the CCT effect on psychological health and cognitive outcomes.

Current evidence suggests that the combination of AE and CCT interventions (COMB) promotes healthy cognitive aging in older adults (Bamidis et al., 2014). Evidence from RCT revealed positive effects from combined training on executive functions (Anderson-Hanley et al., 2012), processing speed (León et al., 2015), memory (Fabre et al., 2002), and general cognitive function (Oswald et al., 2006). However, results differ across trials depending on the type of comparative group and FITT parameters of the intervention (Fabre et al., 2002; Oswald et al., 2006; León et al., 2015). A current hypothesis in reviews is that the combination of AE and CCT may be more efficient than singular interventions (Kraft, 2012; Lauenroth et al., 2016). For example, Ten Brinke et al. (2020) concluded that the combination of 15 min of brisk walking before CCT for 8 weeks provided greater benefits for executive functions, specifically for set shifting. However, more evidence is needed to draw firm conclusions.

Projecte Moviment aims to study the effect of AE, CCT, or COMB on cognition and psychological status in healthy physically inactive older adults (Castells-Sánchez et al., 2019). In this paper, we first addressed our primary objective: to test if AE, CCT, and COMB training—5 times per week for 3 months—improves cognitive performance compared to a control group. Second, we examined whether AE, CCT, and COMB interventions positively impact psychological and subjective daily functioning compared to a control group and if AE and COMB training increase CRF and daily PA compared to the control condition. Finally, we assessed the moderating role of age and sex and the mediating effects of significant changes in PA, CRF, and psychological health in the relationship between the intervention and cognitive benefits.

## Methods

### Study Design

Projecte Moviment is a multi-center, single-blind, proof-of-concept RCT that consisted of four parallel groups undergoing AE, CCT, COMB interventions and a control group during 12 weeks with assessments at baseline and trial completion. The study took place between November 2015 and April 2018. It was developed by the University of Barcelona in collaboration with Institut Universitari d’Investigació en Atenció Primària Jordi Gol, Hospital Germans Trias i Pujol, and Institut Guttmann, and approved by the responsible ethics committees following the Declaration of Helsinki.

The protocol has been published (Castells-Sánchez et al., 2019) and registered in ClinicalTrials.gov (NCT031123900). We synthesize the procedures adhering to the Consolidated Standards of Reporting Trials guidelines.

### Participants

Participants were recruited from the Barcelona metropolitan area using lists of patients of general physicians, volunteers from previous studies, advertisements and oral presentations in health care centers, other local community centers, and local media (newspapers, radio, and TV). Individuals were informed and screened over the phone and in an on-site personal session. If eligible, participants gave written informed consent prior to study commencement.

Participants were eligible if they: (i) were aged between 50 and 70 years; (ii) performed ≤ 120 min/week of PA during last 6 months; (iii) had preserved general cognitive function [Mini-Mental State Examination, MMSE ≥ 24 (Blesa et al., 2001), Montreal Cognitive Assessment 5 min, MoCA 5 min ≥ 6 (Wong et al., 2015)]; (iv) were competent in Catalan or Spanish; and (v) had adequate visual, auditory, and fine motor skills. Participants were excluded if they: (i) participated in any cognitive training program during last 6 months > 2 h/week; (ii) had dementia or mild cognitive impairment diagnosis; (iii) had neurological disorder diagnosis; (iv) had psychiatric diagnosis; (v) scored > 9 in the Geriatric Depression Scale, GDS-15 (Martínez et al., 2002); (vi) consumed psychopharmacological drugs; (vii) had history of drug abuse or alcoholism; (viii) had history of chemotherapy; and (iv) had any contraindication to magnetic resonance imaging. Extended details are included in Castells-Sánchez et al. (2019).

### Randomization

Randomization was performed after the baseline assessments. The allocation sequence was generated by a statistician and it consisted of a random combination of demographic variables that allowed us to balance groups accounting for sex, age, and years of education. Participants were randomly assigned to each condition: AE, CCT, COMB, and control group. The intervention team was responsible for the allocation and the sequence and the group assignment remained blind for the assessors.

### Interventions

The protocol for each intervention condition is explained in more detail elsewhere (Castells-Sánchez et al., 2019). Interventions were applied as individual programs.

#### Aerobic Exercise (AE)

Participants randomized to AE group had to walk briskly, increasing intensity and duration progressively. The first week they had to walk 30 min per day, 5 days per week, up to 9–10 on the Borg Rating of Perceived Exertion Scale (BRPES; Borg, 1982) perceived as light intensity; during the second week, the duration was increased to 45 min and the intensity 9–10 and frequency (5 days per week) were maintained; the following 10 weeks they maintained the duration (45 min) and frequency (5 days per week) and increased the intensity up to 12–14 on BRPES perceived as moderate-high effort.

#### Computerized Cognitive Intervention (CCT)

Participants randomized to CCT group performed a multidomain computerized home-based cognitive training using the Gutmann Neuropersonal Trainer R (GNPT^®^, Spain) (Solana et al., 2014, 2015) in sessions of 45 min, 5 days per week for 12 weeks. Cognitive tasks targeted executive function, visual and verbal memory, and sustained, divided, and selective attention. The GNPT platform calculated an individual profile and adjusted the demand of the tasks depending on the participant level in each domain.

#### Combined Training (COMB)

Participants randomized to COMB group engaged in AE and CCT following the same previously described instructions. AE and CCT were performed separately in single continuous bouts of 45 min for each intervention. They did not have any restriction about the order of the interventions during the day or time-point at which they had to be applied. Therefore, the intervention consisted of 90 min of activity, 5 days per week, for 12 weeks.

#### Control Group

Participants randomized to the control group were on the wait list for 12 weeks and were asked to keep their regular lifestyle.

#### Compliance and Adverse Events

Participants were monitored during the intervention: they received phone calls every 2 weeks, a mid-point visit after 6 weeks of the intervention, and a final visit where they were asked about the level of compliance, interfering events, satisfaction, motivation, and level of difficulty. They registered the frequency of the training and the adverse events occurring during the intervention in a follow-up diary. The AE group was asked to record intensity in which they performed the exercise based on BRPES values. CCT compliance was registered in the software platform too. We ensured that all sources of information about compliance were coherent and allowed us to obtain the level of adherence.

### Outcomes

#### Primary Outcomes

##### Cognitive performance

An extensive neuropsychological battery was designed by Projecte Moviment including standard tests selected for their psychometric qualities and high relevance in the area of study. The neuropsychological battery was administered in at baseline and again within 2 weeks after the completion of the intervention. It was applied before the CRF test or any type of exercise in order to control for the effect of acute exercise on cognitive performance. Tests were performed in a single session of 60–90 min and in the same order for all the participants These tests provided measures of multiple cognitive functions grouped following a theoretical-driven approach (Strauss and Spreen, 1998; Lezak et al., 2012): Flexibility (Trail Making Test B-A time; Tombaugh, 2004), Fluency (letter and category fluency; Peña-Casanova et al., 2009), Inhibition (interference-Stroop Test; Golden, 2001), Working Memory (backward-WAIS-III; Wechsler, 2001), Visuospatial Function (copy accuracy-Rey Osterrieth Complex Figure; Rey, 2009), Language (Boston Naming Test-15; Goodglass et al., 2001), Attention (forward span, digit symbol coding, and symbol search WAIS-III; Wechsler, 2001), Speed (Trail Making Test-A; Tombaugh, 2004; copy time-Rey Osterrieth Complex Figure; Rey, 2009), Visual Memory (memory accuracy-Rey Osterrieth Complex Figure; Rey, 2009), and Verbal Memory (total learning and recall-II Rey Auditory Verbal Learning Test; Schmidt, 1996). Six general domains were designed: (1) Executive Function, (2) Visuospatial Function, (3) Language, (4) Attention-Speed, (5) Memory, and (6) Global Cognitive Function. Extended details are in Supplementary Table 1.

The primary outcome was change in cognitive performance in the assessed cognitive domains. We calculated change from raw data (post-test minus pretest), we obtained z-sample scores for each outcome, and, finally, we averaged z-scores for each cognitive domain and created a global cognitive function score as a sum of all domains.

#### Secondary Outcomes

##### Psychological health and daily activity

We assessed depressive symptoms (GDS-15; Martínez et al., 2002), emotional status (Modified Version of Visual Analog Mood Scale, VAMS; Stern et al., 1997 and Short Informant Questionnaire in Routine Evaluation-Outcome Measure, CORE-OM; Trujillo et al., 2016), sleep quality (Pittsburgh Sleep Quality Index, PSQI; Rico and Fernández, 1997), and subjective performance in daily activities (Short Informant Questionnaire on Cognitive Decline in the Elderly, S-IQCODE; Morales-Gónzalez et al., 1992). Change (post-test minus pretest) was calculated from raw data and used as a secondary outcome.

##### Physical activity

Minnesota Leisure Time PA Questionnaire (VREM; Ruiz et al., 2012) was used to evaluate PA of participants. They reported frequency and duration of the following activities during the last month: sportive walking, sport/dancing, gardening, climbing stairs, shopping walking, and cleaning house. We transformed hours per month into units of metabolic equivalent tasks (METs) estimating the energy expenditure for each category. We calculated Sportive PA (S-PA) and Non-Sportive PA (NS-PA) by adding up the METs spent in different activities and grouping them into the following categories: S-PA—sportive walking and sport/dancing activities—and NS-PA—gardening, climbing stairs, shopping walking, and cleaning house. Change (post-test minus pretest) NS-PA and S-PA was used as a secondary outcome.

##### Cardiorespiratory fitness

The Rockport 1-Mile Test was administered to assess the CRF. Participants walked 1 mile on a treadmill (Technogym^®^, Italy) adjusting their speed in order to be as fast as possible without running. We collected average speed during the test, time to complete the mile, and heart rate once they finished. Maximal aerobic capacity (VO_2_ max) was estimated with the standard equation developed by Kline et al. (1987). Change (post-test minus pretest) in CRF was used as a secondary outcome.

### Statistical Analysis

Statistical procedures were conducted with IBM SPSS Statistics 24. The distribution of raw scores was examined in order to assess data quality (i.e., outliers, skewness). Change (post-test minus pretest) in primary and secondary outcomes was obtained as described above. Baseline comparisons and cross-time partial correlations were performed in order to identify potential confounds.

In order to compare each intervention group to the control group, we performed linear regression models in the intention-to-treat (ITT) and per-protocol (PP) sample using a dummy codification for the *treatment* variable. We regressed change in each cognitive outcome on the baseline outcome score, sex, age, and years of education and the treatment variables (AE vs controls, CCT vs controls, and COMB vs controls) for both ITT and PP samples. Linear regression models for changes in secondary outcomes were executed only in the PP sample.

We used the PROCESS Macro for SPSS (Hayes, 2017) to analyze the moderating effect of individual difference variables—age, sex, and years of education—when the intervention-related changes in cognition were significant.

We applied mediation analyses using the PROCESS Macro when the intervention was related to a significant cognitive change compared to the control group. We analyzed the mediation effect of intervention-related significant changes in secondary outcomes for those primary outcomes where the intervention was significant compared to controls. We introduced a treatment variable (condition vs control) as the independent variable, change in cognition for those functions that showed significant intervention-related changes as the dependent variable, and change in secondary outcomes as mediators controlling for baseline performance score, age, sex, and years of education. These analyses were computed with bias-corrected bootstrap 95% confidence intervals (CIs) based on 5000 bootstrap samples. Significance of mediation was indicated if the CIs in Path AB did not overlap with 0 (Hayes, 2017).

## Results

### Participants

**FIGURE 1:**
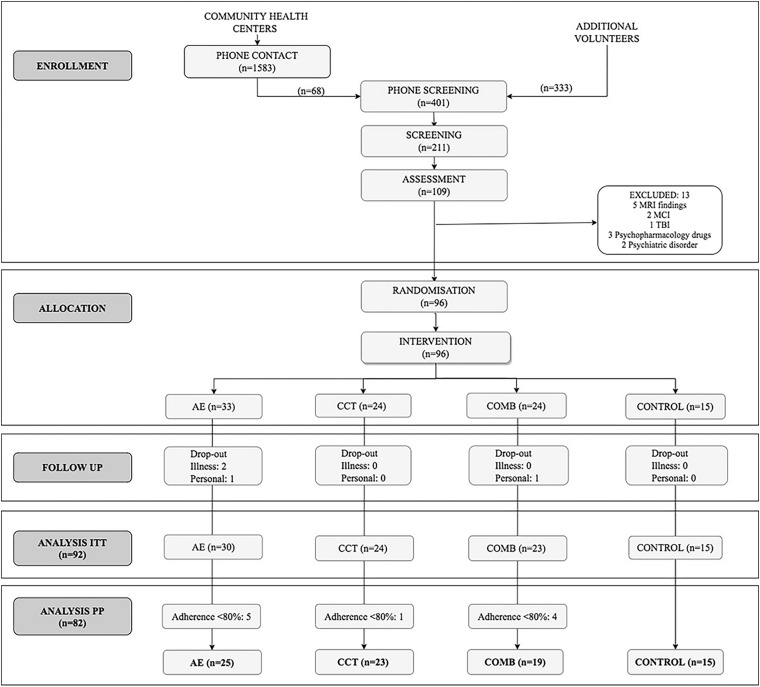
CONSORT flow diagram.

A total of 401 participants were screened by phone and 211 were interviewed in an on-site personal visit (Figure 1). Of the 109 participants who completed baseline assessments, 96 were randomized to the intervention. Only four individuals withdrew from the study for health-related reasons not related to the intervention and for time commitment issues. The ITT sample consisted of 92 participants who completed the intervention and the follow-up assessment (see Supplementary Table 2 for demographic characteristics). Participants with a level of adherence > 80% were included in the PP sample (*n* = 82, 62% female; age = 58.38 ± 5.47). There were not significant differences in demographic characteristics between ITT and PP analyses (see Supplementary Table 3). The PP sample did not show notable differences between groups in demographics (Table 1) nor in cognitive, physical and psychological outcomes at baseline, except in NS-PA and S-IQCODE (extended details in Supplementary Tables 4.1–4.3). Compliance for the aerobic training was 90% for AE group and 90.7% for COMB group while for CCT was 94.1% for CCT group and 91.1% for COMB group. There were no significant differences in levels of compliance between groups. Levels of adherence were not related to sex or age neither. BRPES mean values per week for the AE and COMB are included in Supplementary Table 5.

**TABLE 1:** Participants characteristics at baseline.

	**Total Sample**	**AE**	**CCT**	**COMB**	**Control**	
	**Mean (SD)**	**Mean (SD)**	**Mean (SD)**	**Mean (SD)**	**Mean (SD)**	**Group comparison**
*n* total/*n* females	82/51	25/13	23/16	19/14	15/8	*X*^2^(3) = 3.20, *p* = 0.361
Age (years)	58.38 (5.47)	58.40 (5.12)	57.91 (5.31)	60.32 (5.54)	56.60 (5.97)	*H*(3) = 3.53, *p* = 0.317
Years of education	12.52 (5.57)	12.44 (5.75)	12.04 (4.94)	12.37 (5.43)	13.60 (6.72)	*H*(3) = 0.28, *p* = 0.963
Vocabulary subtest (WAIS-III)	44.14 (8.30)	43.92 (9.53)	44.26 (7.16)	44.53 (8.02)	43.80 (8.98)	*F*(3,77) = 0.03, *p* = 0.993

*Note: AE, aerobic exercise group; CCT, computerized cognitive training group; COMB, combined group; WAIS-III, Wechsler Adult Intelligence Scale. SD: standard deviation X^2^ = chi square; *H* = Kruskall–Wallis *H* test; *F* = Anova test. See Supplementary Tables 4.1–4.3 for more cognitive, physical, and psychological outcomes at baseline.*

### Intervention-Related Changes in Primary Outcomes

Contrasts between each intervention and control group for cognitive outcomes are reported in Table 2. For the PP analysis, results showed a significant improvement in AE compared to Control for Working Memory (SMD = 0.29, *p* = 0.037), Attention (SMD = 0.33, *p* = 0.028), and for the domain of Attention-Speed (SMD = 0.31, *p* = 0.042). There was also a positive, but not significant, effect of this intervention on Fluency (SMD = 0.29, *p* = 0.063) and Speed (SMD = 0.28, *p* = 0.068). The COMB group improved Attention (SMD = 0.30, *p* = 0.043) and Speed (SMD = 0.30, *p* = 0.044) and the domain of Attention-Speed (SMD = 0.30, *p* = 0.041) compared with the Control group. However, there were not significant changes in cognitive outcomes when comparing CTT to the Control group. Results showed greater improvements on Flexibility (SMD = −0.33, *p* = 0.016) in the Control group compared with AE. These analyses revealed similar but less significant results in the ITT sample (see Supplementary Table 6).

**TABLE 2:** Intervention-related changes in primary outcomes.

	**AE vs Controls**	**CCT vs Control**	**COMB vs Control**
	***B* (95%CI),**	***B* (95%CI),**	***B* (95%CI),**
	**SMD, *p*-value**	**SMD, *p*-value**	**SMD, *p*-value**
Executive function	0.16 (−0.14, 0.45), SMD = 0.17, *p* = 0.296	0.03 (−0.27, 0.33), SMD = 0.03, *p* = 0.849	0.15 (−0.16, 0.46), SMD = 0.16, *p* = 0.331
Flexibility	−0.71 (−1.29, −0.13), SMD = −0.33, *p* = 0.016*	−0.23 (−0.81, 0.36), SMD = −0.10, *p* = 0.445	−0.21 (−0.83, 0.42), SMD = −0.09, *p* = 0.509
Fluency	0.42 (−0.02, 0.87), SMD = 0.29, *p* = 0.063	0.13 (−0.33, 0.58), SMD = 0.08, *p* = 0.588	0.14 (−0.34, 0.61), SMD = 0.09, *p* = 0.571
Inhibition	−0.10 (−0.69, 0.50), SMD = −0.04, *p* = 0.749	−0.17 (−0.78, 0.45) SMD = −0.08, *p* = 0.592	0.20 (−0.44, 0.84), SMD = 0.09, *p* = 0.540
Working memory	0.63 (0.04, 1.23), SMD = 0.29, *p* = 0.037*	0.47 (−0.14, 1.09), SMD = 0.21, *p* = 0.127	0.52 (−0.13, 1.17), SMD = 0.22, *p* = 0.113
Visuospatial function	−0.31 (−0.89, 0.27), SMD = −0.14, *p* = 0.294	−0.08 (−0.67, 0.52), SMD = −0.04, *p* = 0.795	−0.14 (−0.77, 0.49), SMD = −0.06, *p* = 0.654
Language	0.11 (−0.43, 0.66), SMD = 0.05, *p* = 0.683	−0.19 (−0.74, 0.37), SMD = −0.09, *p* = 0.503	−0.28 (−0.87, 0.31), SMD = −0.12, *p* = 0.353
Attention-Speed	0.31 (0.01, 0.61), SMD = 0.31, *p* = 0.042*	0.16 (−0.15, 0.46), SMD = 0.15, *p* = 0.316	0.34 (0.01, 0.66), SMD = 0.30, *p* = 0.041*
Attention	0.46 (0.05, 0.88), SMD = 0.33, *p* = 0.028*	0.27 (−0.15, 0.70), SMD = 0.19, *p* = 0.202	0.46 (0.01, 0.90), SMD = 0.30, *p* = 0.043*
Speed	0.28 (−0.02, 0.59), SMD = 0.28, *p* = 0.068	0.13 (−0.18, 0.43) SMD = 0.12, *p* = 0.411	0.33 (0.01, 0.66), SMD = 0.30, *p* = 0.044*
Memory	0.10 (−0.29, 0.49), SMD = 0.08, *p* = 0.602	−0.03 (−0.43, 0.37) SMD = −0.03, *p* = 0.869	−0.04 (−0.46, 0.38), SMD = −0.03, *p* = 0.852
Visual memory	−0.40 (−1.01, 0.20), SMD = −0.19, *p* = 0.189	−0.16 (−0.78, 0.46), SMD = −0.07, *p* = 0.607	0.32 (−0.33, 0.97) SMD = 0.14, *p* = 0.331
Verbal memory	0.39 (−0.11, 0.89), SMD = 0.22, *p* = 0.127	0.07 (−0.45, 0.58), SMD = 0.04, *p* = 0.798	−0.21 (−0.75, 0.33), SMD = −0.11, *p* = 0.444
Global cognitive function	0.12 (−0.07, 0.32), SMD = −0.20, *p* = 0.218	0.02 (−0.18, 0.21) SMD = 0.03, *p* = 0.872	0.12 (−0.09, 0.32), SMD = 0.18, *p* = 0.259

Note: AE, aerobic exercise group; CCT, computerized cognitive training; COMB, combined group; SPA, sportive physical activity; NS-PA, non-sportive physical activity; Total-PA, total physical activity; CRF, cardiorespiratory fitness. SMD = β. Positive SMD values favor AE or CCT or COMB vs Control group. Covariates: sex, age, years of education, and baseline. *p < 0.05.

### Intervention-Related Changes in Secondary Outcomes

Contrasts between each intervention and control group for secondary outcomes are reported in Table 3. The results for psychological health outcomes and daily activity showed no significant improvements in AE and COMB group compared to Control for any outcome. The CCT group showed significant changes in PSQI (SMD = 0.30, *p* = 0.028) compared with the Control group. Results related to PA showed significant improvements for S-PA in AE (*B* = 4515.46, 95% CI: 3611.44, 5419.49) and in COMB (*B* = 4214.04, 95% CI: 3214.97, 5213.12) compared with the Control group. There was also a significant positive change for CRF in AE (*B* = 7.63, 95% CI: 3.93, 11.33) and COMB (*B* = 4.75, 95% CI: 0.73, 8.78) groups. However, as we expected, the CCT group did not improve in PA levels or CRF.

**TABLE 3:** Intervention-related changes in secondary outcomes.

	**AE vs Controls**	**CCT vs Control**	**COMB vs Control**
	***B* (95%CI),**	***B* (95%CI),**	***B* (95%CI),**
	**SMD, *p*-value**	**SMD, *p*-value**	**SMD, *p*-value**
**Phsycological health and daily activity^a^**			
GDS	0.03 (−0.87, 0.93), SMD = 0.01, *p* = 0.940	0.14 (−0.80, 1.08), SMD = 0.04, *p* = 0.769	−0.30 (−1.30, 0.70), SMD = −0.08, *p* = 0.554
VAMS	−0.14 (−0.95, 0.67), SMD = −0.04, *p* = 0.731	−0.21 (−1.03, 0.60), SMD = −0.06, *p* = 0.605	−0.29 (−1.17, 0.58), SMD = −0.08, *p* = 0.508
S-IQCODE	0.66 (−0.83, 2.15), SMD = 0.12, *p* = 0.380	−1.27 (−2.79, 0.26), SMD = −0.22, *p* = 0.101	−0.77 (−2.50, 0.96), SMD = −0.13, *p* = 0.377
PSQI	0.74 (−0.56, 2.04), SMD = 0.15, *p* = 0.261	1.49 (0.16, 2.82), SMD = 0.30, *p* = 0.028*	1.01 (−0.41, 2.43), SMD = 0.19, *p* = 0.161
Total CORE-OM	−1.98 (−5.15, 1.18), SMD = −0.17, *p* = 0.216	−1.17 (−4.44, 2.11), SMD = −0.10, *p* = 0.480	−2.60 (−6.05, 0.86), SMD = −0.20, *p* = 0.139
Well-being CORE-OM	−0.96 (−2.17, 0.26), SMD = −0.23, *p* = 0.123	−0.90 (−2.17, 0.36), SMD = −0.21, *p* = 0.158	−0.58 (−1.90, 0.75), SMD = −0.13, *p* = 0.389
Problems CORE-OM	−1.25 (−2.84, 0.33), SMD = −0.21, *p* = 0.120	−0.40 (−2.02, 1.23), SMD = −0.07, *p* = 0.628	−1.11(−2.85, 0.62), SMD = −0.17, *p* = 0.205
Functioning CORE-OM	0.02 (−1.39, 1.42), SMD = 0.00, *p* = 0.980	0.38 (−1.06, 1.82), SMD = 0.07, *p* = 0.600	−0.73 (−2.26, 0.80), SMD = −0.12, *p* = 0.344
Risk CORE-OM	0.10 (−0.18, 0.38), SMD = 0.09, *p* = 0.473	−0.14 (−0.42, 0.13), SMD = −0.13, *p* = 0.300	−0.07 (−0.37, 0.22), SMD = −0.06, *p* = 0.619
**Physical activity and cardiorespiratory fitness^b^**			
S-PA	4515.46 (3611.44, 5419.49), SMD = 0.83, *p* < 001***	17.07 (−917.10, 951.24), SMD = 0.00, *p* = 0.971	4214.04 (3214.97, 5213.12), SMD = 0.71, *p* < 001***
NS-PA	1797.55 (−1316.63, 4911.72), SMD = 0.17, *p* = 0.254	1947.28 (−1261.49, 5156.04), SMD = 0.17, *p* = 0.230	1864.36 (−1524.03, 5252.74), SMD = 0.16, *p* = 0.276
CRF	7.63 (3.93, 11.33), SMD = 0.49, *p* < 001***	2.12 (−1.59, 5.82), SMD = 0.13, textitp = 0.258	4.75 (0.73, 8.78), SMD = 0.29, *p* = 0.021*

*Note: AE, aerobic exercise group; CCT, computerized cognitive training; COMB, combined group; S-PA, sportive physical activity; NS-PA, non-sportive physical activity; Total-PA, total physical activity; CRF, cardiorespiratory fitness; GDS, Geriatric Depression Scale; VAMS, Modified Version of Visual Analog Mood Scale; S-IQCODE, Short Informant Questionnaire on Cognitive Decline in the Elderly; PSQI, Pittsburg Sleep Quality Index; CORE-OM, Clinical Outcome in Routine Evaluation-Outcome Measure.*

SMD = β. ^*a*^Negative SMD values favor AE or CCT or COMB vs Control group. ^*b*^Positive SMD values favor AE or CCT or COMB vs Control group. Covariates: sex, age, years of education, and baseline. *p < 0.05. ***p < 0.001.

### Intervention-Related Potential Moderators and Mediators

We applied moderation analysis for those cognitive domains that significantly changed with the intervention. The results showed that age and sex did not significantly moderate effects of the intervention on cognitive outcomes. Mediation analyses performed for those primary outcomes that experienced a significant change showed that increases in S-PA significantly mediated the improvements for the domain of Attention-Speed in the AE group (Path C’: *B* = −0.01, SE = 0.23, *p* = 0.973; 95% CI: −0.46, 0.45; Path AB: *B* = 0.31, SE = 0.17, 95% CI: 0.02, 0.68) and in the COMB group (Path C’: *B* = 0.02, SE = 0.23, *p* = 0.926; 95% CI: −0.43, 0.48; Path AB: *B* = 0.29, SE = 0.15, 95% CI: 0.01, 0.62). For the other significant intervention-related cognitive changes, when increases in S-PA were introduced in the model as a mediator, it diminished the association between AE- or COMB- and cognitive changes (direct effects), but indirect effects indicating mediation were not significant. Change in CRF did not significantly mediate the effect of interventions on cognition in any group.

## Discussion

Projecte Moviment is a proof-of-concept RCT that contributes to the understanding of the effects and mechanisms of AE, CCT, or COMB in healthy physically inactive adults aged 50–70 years. In this paper, we addressed our main objectives of the project.

First, we hypothesized that the intervention would improve performance in the assessed cognitive domains compared to a control group. In the PP sample (≥80% adherence), a 12-week 5-days per week AE program showed significant benefits on a measure of Executive Function (Working Memory) and Attention-Speed (Attention) compared to controls. There was also a tendency for positive effects on measures of Fluency and Speed. Our findings concur with previous systematically reviewed literature (Smith et al., 2010; Barha et al., 2017; Northey et al., 2018). We observed these intervention-related changes after 45 h of AE, which is less than the 52 h required to detect cognitive change suggested in previous literature (Gomes-Osman et al., 2018). One plausible explanation about the non-significant results for the Memory domain is that the timeframe of the trial and a late-middle-aged healthy sample could have lead to a ceiling effect in the Memory measures. Another hypothesis is whether a higher frequency, greater intensity, dose, or length of the activity is necessary to observe changes in Memory. It is also possible that changes in Executive Function and Attention-Speed have different time-effects from those related to Memory since they involve different brain areas. As previously published (Weinstein et al., 2012; Hyodo et al., 2016), AE-related improvements in CRF are associated to greater changes in blood flow and metabolic short-term changes and, therefore, greater changes in the prefrontal cortex; area involved with executive and attentional tasks. Interestingly, these findings support the debate about different molecular physiological mechanisms and patterns of AE effects.

Regarding CCT, we did not find a significant transfer effect of CCT to any of the assessed cognitive domains (Lampit et al., 2014) despite the potential improvements in the trained tasks. These results are consistent with other trials that found improvements for the trained tasks but not a generalization of the effects to other untrained objective or subjective measures (Nguyen et al., 2019), which suggest a potential habituation effect rather than a cognitive enhancement. Another possible explanation for the non-significant results is that FITT parameters of the program, such as the frequency, length, or type of activity, may need to be adjusted to observe a significant effect on cognition. As it is suggested by Lampit et al. (2014), the design of the CCT program is a key factor: home-based interventions may not be an effective design and multidomain interventions tend to produce a small effect on cognition as there is not a specific function targeted. However, another hypothesis that should be addressed in future studies is that CCT-related changes could have just produced changes in the structure and function of the brain but not translated into cognitive improvements (Lampit et al., 2015).

In agreement with previous literature (León et al., 2015), a 12-week 5-days per week COMB intervention showed significant positive changes in Attention-Speed, including both subdomains, Attention and Speed, compared to the control condition. There was also a modest, but non-significant positive effect on Working Memory. Our results did not demonstrate greater cognitive benefits when combining AE and CCT as suggested elsewhere (Kraft, 2012; Bamidis et al., 2014; Lauenroth et al., 2016). Null results for General Cognitive Function (Fabre et al., 2002), Executive Function, and Memory (Legault et al., 2011) have also been reported for similar interventions. Our results suggest that COMB-related changes are consistent with the AE-related effects on cognition identified in this study, specifically on Attention, Speed, and Working Memory. More research is needed to better understand how these two types of interventions should be implemented in order to promote greater benefits.

We found a similar pattern but less significant results in the ITT sample, which included all participants that finished the intervention independently of their adherence. This result supports prescribing AE to promote cognitive health and adds support to the relevance of frequency or dose to produce significant changes in cognition as published elsewhere (Gomes-Osman et al., 2018).

We also addressed the effect of AE, CCT, and COMB on our secondary outcomes: psychological health, PA, and CRF. There were no significant changes in psychological health and daily activity outcomes for any intervention group compared to controls except for poorer sleeping quality in CCT compared to the Control group. This result is coherent with previous literature reporting that participants spending the most time in front of the screen showed more probability of sleep problems (Vallance et al., 2015). As expected, we found significant intervention-related changes for S-PA only in the AE and COMB groups compared to controls. Interestingly, and in accordance to other studies (Fabre et al., 2002; Schroeder et al., 2019), AE and COMB had positive effects on cardiovascular health since there was an increase in CRF compared to the control condition. The CCT group did not experience intervention-related changes in the amount of S-PA nor levels of CRF.

Finally, we assessed the moderating effect of individual difference parameters when the intervention had significant effects on cognition compared to control. There were no significant interaction effects for age. This null effect may be explained by a narrow range of age in our sample or a small sample size to detect this interaction. There were also no significant interaction effects for sex. However, as suggested by Barha et al. (2017), positive AE-related effects may be associated with the higher percentage of females in our sample. Second, we analyzed whether significant changes in secondary outcomes mediated the intervention-related changes in cognition. We found that AE- and COMB-related cognitive improvements were mediated by increase in S-PA. Interestingly, despite significant increases in CRF in the AE and COMB groups compared to control, there were no significant mediation effects of CRF for any of the significant intervention-related changes in cognition mentioned above. This result is consistent with previous literature reporting no-significant correlations between change in cognition and change in CRF (Etnier et al., 2006; Young et al., 2015). Moreover, previous findings suggest that CRF may be a mediator of cognitive change related to AE only in samples aged ≥ 70 years but not in younger adults (Bherer et al., 2019). The remaining question is whether CRF change is a mediator only when the physiological mechanisms (e.g., oxidative stress, immune system molecules, etc.) of reparation are damaged by normal age-decline or pathology. In addition, since there are sex differences in the physiological adaptations to AE (Barha and Liu-Ambrose, 2018) and in the CRF level across lifespan (Al-Mallah et al., 2016), it would be interesting to assess the mediating effect of CRF stratifying results by sex in larger samples. These findings suggest that several physiological molecular correlates and individual variables influenced by FITT parameters, apart from CRF, may play an important role in the described benefits.

Our multidomain assessment allowed us to widely assess the effects of these interventions in a novel short-term high frequency design of the interventions. Moreover, we could assess the role of individual variables as well as the potential mediating effect of CRF and energy expenditure in S-PA. Despite the sample size allowed us to obtain results in our main aims that are coherent with the literature (Colcombe and Kramer, 2003), the number of participants in each group did not allow to perform intra-group analyses by sex or age. The higher percentage of females in our sample might influence our results as suggested in previous literature (Barha et al., 2017) as well as the wait-list control group which participants might have reduced treatment expectations. We also acknowledge that adherence is based on self-reported information and should be objectively monitored in future studies in order to correct potential desirability bias. The low level of attrition and the stringent inclusion criteria lead to a very healthy motivated sample, which helped to contribute to the research field with a rigorous sample but could have promoted a ceiling effect in the analyses, despite the statistical corrections applied. Our study involved individually applied interventions. Further studies should address and compare the effect of these interventions when they are applied individually or in a group given the recent bibliography suggesting cognitive enhancement as a result of social interaction (Kelly et al., 2017). These facts should be considered when translating those results into clinical practice where the population is more diverse.

## Conclusion

Projecte Moviment adds scientific support to the clinical relevance of lifestyle interventions in the promotion of cognitive health. In this proof-of-concept trial, we conclude that AE applied as walking, and performed 45 h in a 12-weeks, 5-days per week program, may provide cognitive benefits for Executive function and Attention-Speed, and that the combination with CCT may lead to similar results in healthy adults aged 50–70 years.

Our results open the debate of the potential different effects and physiological mechanisms of these interventions on each cognitive function and highlight the importance of frequency and dose of the activity.

## Data Availability Statement

The raw data supporting the conclusions of this article will be made available by the authors, without undue reservation.

## Ethics Statement

The studies involving human participants were reviewed and approved by the Bioethics Commission of the University of Barcelona (IRB00003099) Clinical Research Ethics Committee of IDIAP Jordi Gol (P16/181). The patients/participants provided their written informed consent to participate in this study.

## Author Contributions

MM conceptualized the study, contributed to the study design and implementation as principal investigator, and also guided and supervised all the statistical analysis and writing of this manuscript. PT-M and KE made substantial contributions to the design and implementation of the trial. AC-S and FR-C contributed to the design and implementation of this trial, recruited the participants and evaluated them before and after interventions, also analyzed the data, and wrote this manuscript. NL-V collaborated with recruitment and did the follow-up of the intervention groups. AG-M and JT assisted in the use of GNPT program for computerized cognitive training. GP guided and supervised the statistical analysis. PM-A, MA, RD-A, JS-R, and CC contributed to the implementation of the trial from their area of expertise. All authors reviewed the manuscript and provided final approval for publication of the content.

## Conflict of Interest

The authors declare that the research was conducted in the absence of any commercial or financial relationships that could be construed as a potential conflict of interest.
