# Supplementary material for: Effects of Aerobic Exercise, Cognitive and Combined Training on Cognition in Physically Inactive Healthy Late-Middle-Aged Adults: The Projecte Moviment Randomized Controlled Trial
Source: Front Aging Neurosci. 2020 Oct 29;12:590168. doi: 10.3389/fnagi.2020.590168 (PMC7664521; doi:10.3389/fnagi.2020.590168)
Supplement: Supplementary file 1 [file Table_1.DOCX]

| **Table 1. Cognitive outcomes: variables and measures** | | | |
| --- | --- | --- | --- |
| **Composites 1^st^ Level** | **Composites 2^nd^ Level** | **Tests - Subtest** | **Measure** |
| Executive Function | Flexibility | TMT B -A | Z score |
|  | Fluency | Letter fluency | Z score |
|  |  | Category fluency | Z score |
|  | Inhibition | Stroop - Interference | Z score |
|  | Working Memory | WAIS III - Backward Span | Z score |
| Visuospatial Function | Visuospatial Function | ROCF - Copy Accuracy | Z score |
| Language | Language | BNT (15 items) | Z score |
| Attention - Speed | Attention | WAIS III - Forward Span | Z score |
|  |  | WAIS III - Digit Symbol Coding | Z score |
|  |  | WAIS-III - Symbol Search | Z score |
|  | Speed | TMT - A | Z score |
|  |  | ROCF - Copy Time | Z score |
| Memory | Visual Memory | ROCF - Memory Accuracy | Z score |
|  | Verbal Memory | RAVLT - Total Learning | Z score |
|  |  | RAVLT - Recall II | Z score |
| *Note: TMT, Trail Making Test (Tombaugh, 2004); Verbal Fluency Tests (Peña-Casanova et al., 2009); Stroop Test (Golden, 2001); WAIS-III, Wechsler Adult Intelligence Scale (Wechsler, 2001); ROCF, Rey-Osterrieth Complex Figure (Rey, 2009); BNT, Boston Naming Test (Goodglass et al., 2001); RAVLT, Rey Auditory Verbal Learning Test (Schmidt, 1996).* | | | |
