# Supplementary material for: Effects of Aerobic Exercise, Cognitive and Combined Training on Cognition in Physically Inactive Healthy Late-Middle-Aged Adults: The Projecte Moviment Randomized Controlled Trial
Source: Front Aging Neurosci. 2020 Oct 29;12:590168. doi: 10.3389/fnagi.2020.590168 (PMC7664521; doi:10.3389/fnagi.2020.590168)
Supplement: Supplementary file 2 [file Table_2.DOCX]

| Table 2. Participants Characteristics in the ITT sample at Baseline | | | | | | |
| --- | --- | --- | --- | --- | --- | --- |
|  | **Total**  **Mean (SD)** | **AE**  **Mean (SD)** | **CCT**  **Mean (SD)** | **COMB**  **Mean (SD)** | **Control**  **Mean (SD)** | **Comparison Group**  **Mean (SD)** |
| n total / n females | 92 / 58 | 30 / 16 | 24 / 17 | 23 / 17 | 15 / 8 | *Χ^2^*(3) = 3.61, *p* = .306 |
| Age (years) | 57.91 (5.50) | 57.90 (5.22) | 57.63 (5.38) | 59.09 (5.79) | 56.60 (5.97) | H(3) = 1.66, *p* = .645 |
| Years of education | 12.76 (5.40) | 13.15 (5.56) | 12.04 (4.83) | 12.43 (5.04) | 13.60 (6.72) | H(3) = 1.05, *p* = .788 |
| Vocabulary subtest (WAIS-II) | 44.04 (8.02) | 43.59 (8.91) | 43.88 (7.26) | 44.96 (7.40) | 43.80 (8.98) | F(3,87) = 0.14, *p* = .939 |
| *Note: AE = Aerobic Exercise group; CCT = Computerized Cognitive Training group; COMB = Combined group; WAIS-III = Wechsler Adult Intelligence Scale X2 = chi square; H= Kruskall Wallis H test; F= Anova test* | | | | | | |
