# Supplementary material for: Effects of Aerobic Exercise, Cognitive and Combined Training on Cognition in Physically Inactive Healthy Late-Middle-Aged Adults: The Projecte Moviment Randomized Controlled Trial
Source: Front Aging Neurosci. 2020 Oct 29;12:590168. doi: 10.3389/fnagi.2020.590168 (PMC7664521; doi:10.3389/fnagi.2020.590168)
Supplement: Supplementary file 4 [file Table_4.DOCX]

| **Table 4.1. Group comparison at Baseline: z-scores of Cognitive Domains** | | | | | |
| --- | --- | --- | --- | --- | --- |
| **Variables** | **Groups** | **n** | **Mean** | **SD** | **ANOVA / H de Kruskall Wallis** |
|  |  |  |  |  |  |
| Executive Function | AE | 24 | -0.01 | 0.72 | F(3,75) = 0.81, *p* = .492 |
|  | CCT | 23 | 0.16 | 0.60 |  |
|  | COMB | 19 | -0.08 | 0.50 |  |
|  | Control | 13 | -0.15 | 0.81 |  |
| Flexibility | AE | 25 | 0.10 | 1.05 | H(3) = 1.53, *p* = .676 |
|  | CCT | 23 | -0.10 | 1.06 |  |
|  | COMB | 19 | -0.03 | 0.80 |  |
|  | Control | 14 | 0.04 | 1.12 |  |
| Fluency | AE | 25 | 0.02 | 0.91 | F(3,77) = 1.19, *p* = .318 |
|  | CCT | 23 | 0.22 | 0.76 |  |
|  | COMB | 19 | -0.10 | 0.82 |  |
|  | Control | 14 | -0.28 | 0.80 |  |
| Inhibition | AE | 24 | -0.06 | 1.03 | F(3,77) = 1.18, *p* = .323 |
|  | CCT | 23 | 0.32 | 0.86 |  |
|  | COMB | 19 | -0.16 | 1.18 |  |
|  | Control | 15 | -0.20 | 0.89 |  |
| Working Memory | AE | 25 | -0.06 | 1.10 | H(3) = 1.29, *p* = .732 |
|  | CCT | 23 | 0.14 | 1.05 |  |
|  | COMB | 19 | -0.01 | 0.69 |  |
|  | Control | 15 | -0.11 | 1.16 |  |
| Visuospatial Function | AE | 25 | 0.14 | 0.93 | H(3) = 3.40, *p* = .334 |
|  | CCT | 23 | -0.20 | 1.04 |  |
|  | COMB | 19 | -0.19 | 1.24 |  |
|  | Control | 15 | 0.32 | 0.62 |  |
| Language | AE | 25 | 0.01 | 1.09 | H(3) = 0.71, *p* = .870 |
|  | CCT | 23 | -0.04 | 1.08 |  |
|  | COMB | 19 | -0.10 | 0.91 |  |
|  | Control | 15 | 0.16 | 0.89 |  |
| Attention-Speed | AE | 24 | -0.02 | 1.01 | H(3) = 2.34, *p* = .506 |
|  | CCT | 23 | 0.13 | 0.65 |  |
|  | COMB | 19 | -0.17 | 0.68 |  |
|  | Control | 14 | 0.09 | 0.44 |  |
| Attention | AE | 25 | 0.03 | 0.95 | F(3,77) = 0.70, *p* = .553 |
|  | CCT | 23 | 0.15 | 0.71 |  |
|  | COMB | 19 | -0.20 | 0.76 |  |
|  | Control | 14 | 0.03 | 0.62 |  |
| Speed | AE | 24 | -0.12 | 1.21 | H(3) = 1.64, *p* = .650 |
|  | CCT | 23 | 0.11 | 0.77 |  |
|  | COMB | 19 | -0.12 | 0.74 |  |
|  | Control | 15 | 0.17 | 0.32 |  |
| Memory | AE | 25 | 0.03 | 0.70 | H(3) = 0.95, *p* = .815 |
|  | CCT | 23 | 0.13 | 0.66 |  |
|  | COMB | 19 | -0.14 | 0.92 |  |
|  | Control | 14 | -0.09 | 0.95 |  |
| Visual Memory | AE | 25 | 0.17 | 1.05 | F(3,78) = 1.00, *p* =.398 |
|  | CCT | 23 | 0.06 | 0.98 |  |
|  | COMB | 19 | -0.34 | 1.17 |  |
|  | Control | 15 | 0.04 | 0.65 |  |
| Verbal Memory | AE | 25 | -0.04 | 0.71 | H(3) = 0.70, *p* = .874 |
|  | CCT | 23 | 0.16 | 0.79 |  |
|  | COMB | 19 | -0.04 | 1.08 |  |
|  | Control | 14 | -0.14 | 1.29 |  |
| Global Cognitive Function | AE | 23 | 0.01 | 0.73 | F(3,74) = 0.51, *p* = .675 |
|  | CCT | 23 | .011 | 0.56 |  |
|  | COMB | 19 | -0.13 | 0.57 |  |
|  | Control | 13 | -0.03 | 0.59 |  |
| *Note: AE = Aerobic exercise group; CCT = Computerized Cognitive Training group; COMB = Combined group.* | | | | | |

| **Table 4.2. Group comparison at Baseline: Psychological Health and Daily Activity** | | | | | |
| --- | --- | --- | --- | --- | --- |
| **Variables** | **Groups** | **N** | **Mean** | **SD** | **ANOVA / H de Kruskall Wallis** |
|  |  |  |  |  |  |
| GDS | AE | 25 | 1.24 | 1.72 | H(3) = 6.18, *p* = .103 |
|  | CCT | 22 | 2.14 | 2.57 |  |
|  | COMB | 19 | 2.47 | 1.90 |  |
|  | Control | 15 | 1.27 | 1.16 |  |
| VAMS | AE | 25 | 2.44 | 1.33 | H(3) = 2.55, *p* = .467 |
|  | CCT | 23 | 3.22 | 2.09 |  |
|  | COMB | 19 | 2.79 | 2.25 |  |
|  | Control | 15 | 3.27 | 2.19 |  |
| S-IQCODE | AE | 25 | 52.00 | 2.69 | H(3) = 12.94, *p* = .005 |
|  | CCT | 23 | 52.44 | 2.48 |  |
|  | COMB | 19 | 55.69 | 4.79 |  |
|  | Control | 15 | 51.87 | 2.30 |  |
| PSQI | AE | 25 | 4.24 | 2.74 | H(3) = 0.55, *p* = .909 |
|  | CCT | 23 | 4.30 | 2.64 |  |
|  | COMB | 19 | 4.84 | 2.99 |  |
|  | Control | 15 | 4.47 | 2.30 |  |
| Total  CORE-OM | AE | 25 | 12.92 | 6.81 | H(3) = 1.95, *p* = .583 |
|  | CCT | 23 | 15.65 | 9.03 |  |
|  | COMB | 19 | 14.90 | 8.53 |  |
|  | Control | 15 | 11.40 | 6.01 |  |
| Well-being  CORE-OM | AE | 25 | 3.92 | 2.02 | H(3) = 5.98, *p* = .113 |
|  | CCT | 23 | 4.70 | 3.15 |  |
|  | COMB | 19 | 4.32 | 2.67 |  |
|  | Control | 15 | 2.60 | 1.64 |  |
| Problems  CORE-OM | AE | 25 | 4.20 | 3.22 | H(3) = 2.90, *p* = .407 |
|  | CCT | 23 | 5.44 | 3.62 |  |
|  | COMB | 19 | 5.68 | 3.74 |  |
|  | Control | 15 | 4.33 | 2.38 |  |
| Functioning  CORE-OM | AE | 25 | 4.76 | 2.89 | H(3) = 1.37, *p* = .714 |
|  | CCT | 23 | 5.26 | 3.41 |  |
|  | COMB | 19 | 4.47 | 3.36 |  |
|  | Control | 15 | 4.13 | 2.50 |  |
| Risk  CORE-OM | AE | 25 | 0.02 | 0.22 | H(3) = 5.33, *p* = .149 |
|  | CCT | 23 | 0.26 | 0.62 |  |
|  | COMB | 19 | 0.16 | 0.38 |  |
|  | Control | 15 | 0.33 | 0.62 |  |
|  | Control | 15 | 11.40 | 6.01 |  |
| *Note: AE = Aerobic exercise group; CCT = Computerized Cognitive Training group; COMB = Combined group; GDS=Geriatric Depression Scale; VAMS = Modified Version of Visual Analog Mood Scale; S-IQCODE = Short Informant Questionnaire on Cognitive Decline in the Elderly; PSQI = Pittsburg Sleep Quality Index; CORE-OM = Clinical Outcome in Routine Evaluation-Outcome Measure* | | | | | |

| **Table 4.3. Group Comparison at Baseline: PA and CRF** | | | | | |
| --- | --- | --- | --- | --- | --- |
| **Variables** | **Groups** | **N** | **Mean** | **SD** | **ANOVA / H de Kruskall Wallis** |
|  |  |  |  |  |  |
| CRF | AE | 19 | 25.25 | 10.16 | F(3,67) = 1.08, *p* = .362 |
|  | CCT | 20 | 26.11 | 12.50 |  |
|  | COMB | 17 | 27.34 | 8.75 |  |
|  | Control | 15 | 20.65 | 12.69 |  |
| S-PA | AE | 25 | 451.98 | 699.40 | H(3) = 2.92, *p* = .404 |
|  | CCT | 23 | 439.83 | 713.63 |  |
|  | COMB | 19 | 778.79 | 908.77 |  |
|  | Control | 15 | 366.80 | 618.17 |  |
| NS-PA | AE | 25 | 5595.73 | 3918.34 | H(3) = 7.96, *p* = .047 |
|  | CCT | 23 | 9113.74 | 7104.64 |  |
|  | COMB | 19 | 10295.68 | 6159.04 |  |
|  | Control | 15 | 7038.40 | 6628.45 |  |
| *Note: AE = Aerobic exercise group; CCT = Computerized Cognitive Training group; COMB = Combined group; S-PA = Sportive Physical Activity; NS-PA = Non Sportive Physical Activity; Total-PA = Total Physical Activity; CRF = Cardiorespiratory Fitness.* | | | | | |
