# Supplementary material for: Effects of Aerobic Exercise, Cognitive and Combined Training on Cognition in Physically Inactive Healthy Late-Middle-Aged Adults: The Projecte Moviment Randomized Controlled Trial
Source: Front Aging Neurosci. 2020 Oct 29;12:590168. doi: 10.3389/fnagi.2020.590168 (PMC7664521; doi:10.3389/fnagi.2020.590168)
Supplement: Supplementary file 5 [file Table_5.DOCX]

| **Table 5. BRPES means values for AE and COMB group** | | | |
| --- | --- | --- | --- |
| Weeks of program | **AE group**  Mean (SD) | **COMB group**  Mean (SD) |  |
| 1 week | 10.03 (0.94) | 10.10 (1.45) |  |
| 2 week | 10.49 (1.20) | 11.10 (1.77) |  |
| 3 week | 12.41 (1.19) | 12.54 (1.32) |  |
| 4 week | 12.68 (1.29) | 12.72 (1.19) |  |
| 5 week | 12.86 (0.92) | 12.74 (1.41) |  |
| 6 week | 13.10 (0.83) | 13.05 (1.18) |  |
| 7 week | 12.69 (1.22) | 13.14 (1.13) |  |
| 8 week | 12.86 (1.04) | 12.71 (1.55) |  |
| 9 week | 13.04 (0.88) | 12.94 (1.30) |  |
| 10 week | 13.03 (0.90) | 13.03 (1.40) |  |
| 11 week | 12.98 (0.95) | 13.17 (1.32) |  |
| 12 week | 13.06 (0.96) | 13.31 (1.32) |  |
