# Supplementary material for: Effects of Aerobic Exercise, Cognitive and Combined Training on Cognition in Physically Inactive Healthy Late-Middle-Aged Adults: The Projecte Moviment Randomized Controlled Trial
Source: Front Aging Neurosci. 2020 Oct 29;12:590168. doi: 10.3389/fnagi.2020.590168 (PMC7664521; doi:10.3389/fnagi.2020.590168)
Supplement: Supplementary file 6 [file Table_6.DOCX]

| **Table 6. Differences in change in cognitive domains between interventions and control group in ITT sample** | | | |
| --- | --- | --- | --- |
|  | AE vs Controls  B (95%CI),  SMD, p | CCT vs Control  B (95%CI),  SMD, p | COMB vs Control  B (95%CI),  SMD, p |
| Executive Function | 0.13 (-0.15, 0.41),  SMD = 0.14, *p* = .370 | 0.03 (-0.26, 0.32),  SMD = 0.03, *p* = .847 | 0.13 (-0.17, 0.42),  SMD = 0.13, *p* = .401 |
| Flexibility | -0.65 (-1.21, -0.08),  SMD = -0.31, *p* = .026* | -0.21 (-0.80, 0.38),  SMD = -0.09, *p* = .476 | -0.23 (-0.83, 0.37),  SMD = -0.10, *p* = .444 |
| Fluency | 0.44 (0.01, 0.88),  SMD = 0.29, *p* = .047* | 0.12 (-0.34, 0.58),  SMD = 0.08, *p* = .608 | 0.24 (-0.22, 0.71),  SMD = 0.15, *p* = .299 |
| Inhibition | -0.19 (-0.77, 0.39),  SMD = -0.09, *p* = .511 | -0.14 (-0.75, 0.47),  SMD = -0.06, *p* = .642 | 0.13 (-0.49, 0.74),  SMD = 0.06, *p* = .678 |
| Working Memory | 0.54 (-0.04, 1.12),  SMD = 0.26, *p* = .066 | 0.45 (-0.16, 1.06),  SMD = 0.20, *p* = .148 | 0.39 (-0.23, 1.01),  SMD = 0.17, *p* = .212 |
| Visuospatial Function | -0.23 (-0.78, 0.33),  SMD = -0.11, *p* = .420 | -0.10 (-0.69, 0.49),  SMD = -0.05, *p* = .730 | -0.32 (-0.91, 0.28),  SMD = -0.14, *p* = .294 |
| Language | 0.03 (-0.47, 0.53),  SMD = 0.01, *p* = .908 | -0.18 (-0.71, 0.34),  SMD = -0.08, *p* = .495 | -0.29 (-0.82, 0.25),  SMD = -0.13, *p* = .291 |
| Attention-Speed | 0.27 (-0.02, 0.56),  SMD = 0.27, *p* = .063 | 0.14 (-0.16, 0.44),  SMD = 0.14, *p* = .347 | 0.29 (-0.01, 0.60),  SMD = 0.27, *p* = .058 |
| Attention | 0.39 (-0.00, 0.78),  SMD = 0.29, *p* = .051 | 0.25 (-0.16, 0.66),  SMD = 0.18, *p* = .225 | 0.38 (-.04, 0.79),  SMD = 0.26, *p* = .075 |
| Speed | 0.05 (-0.34, 0.44),  SMD = 0.03, *p* = .803 | 0.04 (-0.36, 0.45),  SMD = 0.03, *p* =.831 | 0.18 (-0.24, 0.59),  SMD = 0.11, *p* = .399 |
| Memory | 0.14 (-0.24, 0.51),  SMD = 0.11, *p* = .471 | -0.04 (-0.43, 0.35),  SMD = -0.03, *p* = .837 | -0.06 (-0.46, 0.33),  SMD = -0.05, *p* = .747 |
| Visual memory | -0.24 (-0.83, 0.35),  SMD = -0.11, *p* = .418 | -0.19 (-0.81, 0.43),  SMD = -0.08, *p* =.547 | 0.11 (-0.52, 0.73),  SMD = 0.05, *p* = .739 |
| Verbal Memory | 0.34 (-0.14, 0.82),  SMD = 0.20, *p* = .165 | 0.05 (-0.45, 0.56),  SMD = 0.03, *p* =.840 | -0.15 (-0.66, 0.36),  SMD = -0.08, *p* = .560 |
| Global Cognitive Function | 0.13 (-0.06, 0.32),  SMD = 0.22, *p* = .166 | 0.02 (-0.17, 0.22),  SMD = 0.04, *p* =.824 | 0.09 (-0.11, 0.28),  SMD = 0.13, *p* = .384 |
| *Note: AE = Aerobic Exercise group; CCT = Computerized Cognitive Training; COMB = Combined Group; S-PA = Sportive Physical Activity; NS-PA = Non Sportive Physical Activity; Total-PA = Total Physical Activity; CRF = Cardiorespiratory Fitness*  *Covariates: sex, age, years of education and Baseline score*  *SMD = β; Positive SMD values favor AE or CCT or COMB vs Control group; *p < .05; **p < .01* | | | |
